# *N*-(1-Allyl-1*H*-indazol-5-yl)-4-methyl­benzene­sulfonamide

**DOI:** 10.1107/S1600536813032091

**Published:** 2013-11-30

**Authors:** Hakima Chicha, El Mostapha Rakib, Hafid Abderrafia, Mohamed Saadi, Lahcen El Ammari

**Affiliations:** a Laboratoire de Chimie Organique et Analytique, Université Sultan Moulay Slimane, Faculté des Sciences et Techniques, Béni-Mellal, BP 523, Morocco; b Laboratoire de Chimie du Solide Appliquée, Faculté des Sciences, Université Mohammed V-Agdal, Avenue Ibn Battouta, BP. 1014, Rabat, Morocco

## Abstract

The asymmetric unit of the title compound, C_17_H_17_N_3_O_2_S, contains two independent mol­ecules linked by an N—H⋯O hydrogen bond. The mol­ecules show different conformations. In the first mol­ecule, the fused five- and six-membered ring system is almost perpendicular to the plane through the atoms forming the allyl group, as indicated by the dihedral angle of 85.1 (4)°. The dihedral angle with the methyl­benzene­sulfonamide group is 78.8 (1)°. On the other hand, in the second mol­ecule, the dihedral angles between the indazole plane and the allyl and methyl­benzene­sulfonamide groups are 80.3 (3) and 41.5 (1)°, respectively. In the crystal, mol­ecules are further linked by N—H⋯N and C—H⋯O hydrogen bonds, forming a three-dimensional network.

## Related literature

For the biological activity of sulfonamides, see: Bouissane *et al.* (2006 ▶); El-Sayed *et al.* (2011 ▶); Mustafa *et al.* (2012 ▶). For similar compounds, see: Abbassi *et al.* (2012 ▶, 2013 ▶); Chicha *et al.* (2013 ▶).
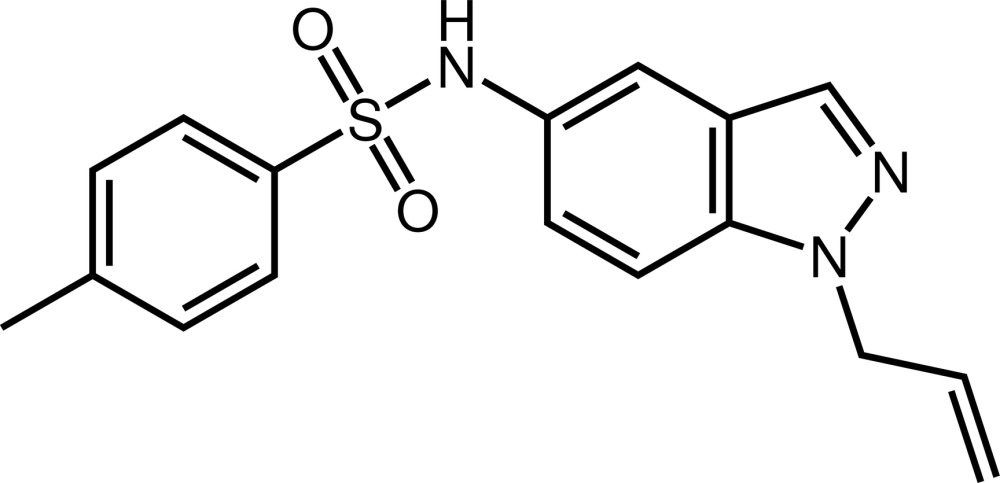


## Experimental

### 

#### Crystal data

C_17_H_17_N_3_O_2_S
*M* *_r_* = 327.40
Triclinic, 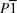

*a* = 8.8200 (4) Å
*b* = 10.4769 (5) Å
*c* = 19.7407 (10) Å
α = 80.211 (1)°
β = 78.984 (1)°
γ = 69.784 (1)°
*V* = 1669.51 (14) Å^3^
*Z* = 4
Mo *K*α radiation
μ = 0.21 mm^−1^
*T* = 296 K
0.42 × 0.35 × 0.28 mm

#### Data collection

Bruker X8 APEX diffractometer
Absorption correction: multi-scan (*SADABS*; Bruker, 2009 ▶) *T*_min_ = 0.693, *T*_max_ = 0.747
31703 measured reflections
6791 independent reflections
5368 reflections with *I* > 2σ(*I*)
*R*_int_ = 0.031

#### Refinement

*R*[*F* ^2^ > 2σ(*F* ^2^)] = 0.042
*wR*(*F* ^2^) = 0.120
*S* = 1.02
6791 reflections
415 parameters
H-atom parameters constrained
Δρ_max_ = 0.32 e Å^−3^
Δρ_min_ = −0.28 e Å^−3^

### 

Data collection: *APEX2* (Bruker, 2009 ▶); cell refinement: *SAINT* (Bruker, 2009 ▶); data reduction: *SAINT*; program(s) used to solve structure: *SHELXS97* (Sheldrick, 2008 ▶); program(s) used to refine structure: *SHELXL97* (Sheldrick, 2008 ▶); molecular graphics: *ORTEP-3 for Windows* (Farrugia, 2012 ▶); software used to prepare material for publication: *PLATON* (Spek, 2009 ▶) and *publCIF* (Westrip, 2010 ▶).

## Supplementary Material

Crystal structure: contains datablock(s) I. DOI: 10.1107/S1600536813032091/im2445sup1.cif

Structure factors: contains datablock(s) I. DOI: 10.1107/S1600536813032091/im2445Isup2.hkl

Supplementary material file. DOI: 10.1107/S1600536813032091/im2445Isup3.cml

Additional supplementary materials:  crystallographic information; 3D view; checkCIF report

## Figures and Tables

**Table 1:** Hydrogen-bond geometry (Å, °)

*D*—H⋯*A*	*D*—H	H⋯*A*	*D*⋯*A*	*D*—H⋯*A*
N6—H6*N*⋯O1	0.79	2.11	2.900 (2)	176
N3—H3*N*⋯N5^i^	0.80	2.19	2.983 (2)	175
C21—H21⋯O4^ii^	0.93	2.49	3.245 (2)	138
C7—H7⋯O2^iii^	0.93	2.48	3.358 (2)	158

Symmetry codes: (i) 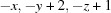; (ii) 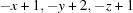; (iii) 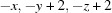.

**Fig. 1.**
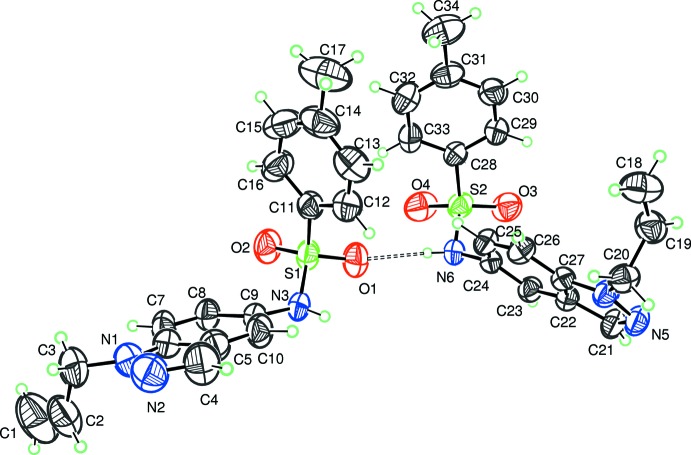
The two molecules building the asymmetric unit of the title compound with the atom-labelling scheme. Displacement ellipsoids are drawn at the 50% probability level. H atoms are represented as small circles.

**Fig. 2.**
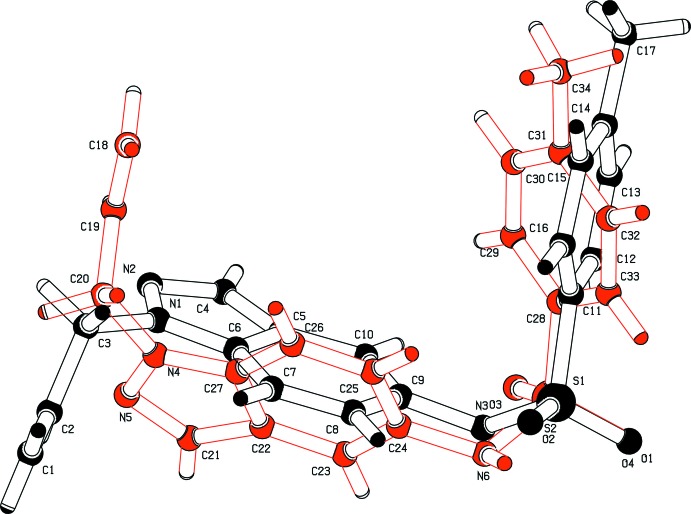
Automatic fit of the two crystallographically independent molecules.

## supplementary crystallographic information

### 1. Comment

Sulfonamides are an important class of compounds which are widely used in the design of diverse classes of drug candidates. These compounds exhibit a wide range of biological activities such as anticancer, anti-inflammatory, and antiviral functions (Bouissane, *et al.*, 2006; El-Sayed, *et al.*, 2011; Mustafa, *et al.* 2012). The present work is a continuation of the investigation of sulfonamides derivatives published recently by our team (Abbassi, *et al.*, 2012; Abbassi, *et al.*, 2013; Chicha, *et al.*, 2013).

The two independent molecules forming the asymmetric unit of the title compound are linked by a weak hydrogen bond (C13–H13···O4) and have different conformations as shown in Fig.1. Each molecule is built up from fused five- and six-membered rings linked to a methylbenzenesulfonamide and allyl groups. In the first molecule, the indazole ring system makes dihedral angles of 78.8 (1)° and 85.1 (4)° with the plane through the methylbenzenesulfonamide group and that through the allyl group, respectively. In the second molecule, the indazole system is almost perpendicular to the plane through the atoms forming the allyl group, as indicated by the dihedral angle of 80.3 (3)°. The dihedral angle between the indazole ring and the benzene ring belonging to the methylbenzenesulfonamide is 41.5 (1)°. In addition, the most important difference between the two conformations of molecules is the orientation of their allyl substituents (Fig.2). In the crystal, molecules are linked by N—H···N, N—H···O and C—H···O hydrogen bonds, forming a three-dimensional network (Table 1).

### 2. Experimental

A mixture of 1-allyl-5-nitroindazole (250 mg, 1.22 mmol) and anhydrous SnCl_2_ (1.1 g, 6.1 mmol) in 25 ml of absolute ethanol was heated to 333 K for 6 h. After reduction, the starting material disappeared, and the solution was allowed to cool down. The pH was made slightly basic (pH 7–8) by addition of 5% aqueous potassium bicarbonate before extraction with ethyl acetate. The organic phase was washed with brine and dried over magnesium sulfate. The solvent was removed to afford the amine, which was immediately dissolved in pyridine (5 ml) and then reacted with 4-methylbenzenesulfonyl chloride (240 mg, 1.25 mmol) at room temperature for 24 h. After the reaction mixture was concentrated *in vacuo*, the resulting residue was purified by flash chromatography (eluted with ethyl acetate: hexane 1:9). The title compound was recrystallized from ethanol at room temperature (yield: 75%, m.p. 374 K).

### 3. Refinement

H atoms were located in a difference map and treated as riding with N—H = 0.89 Å, C–H = 0.96 Å (methyl), C–H = 0.97 Å (methylene) and C–H = 0.93 Å (aromatic CH, terminal =CH_2_), respectively. All hydrogen atoms were refined using a riding model with *U*_iso_(H) = 1.5 *U*_eq_(C) for methyl groups and *U*_iso_(H) = 1.2 *U*_eq_(C,N) for all other hydrogen atoms.

Values of atomic displacements of C1, C2, C14 and C15 which belong to the first molecule (S1, O1, O2, N1 - N3, C1 - C17) and are larger than those observed in the second molecule for corresponding carbons.

### Crystal data

**Table:**

C_17_H_17_N_3_O_2_S	*Z* = 4
*M**_r_* = 327.40	*F*(000) = 688
Triclinic, *P*1	*D*_x_ = 1.303 Mg m^−^^3^
Hall symbol: -p 1	Mo *K*α radiation, λ = 0.71073 Å
*a* = 8.8200 (4) Å	Cell parameters from 6791 reflections
*b* = 10.4769 (5) Å	θ = 2.4–26.4°
*c* = 19.7407 (10) Å	µ = 0.21 mm^−^^1^
α = 80.211 (1)°	*T* = 296 K
β = 78.984 (1)°	Block, colourless
γ = 69.784 (1)°	0.42 × 0.35 × 0.28 mm
*V* = 1669.51 (14) Å^3^	

### Data collection

**Table:**

Bruker X8 APEX diffractometer	6791 independent reflections
Radiation source: fine-focus sealed tube	5368 reflections with *I* > 2σ(*I*)
Graphite monochromator	*R*_int_ = 0.031
φ and ω scans	θ_max_ = 26.4°, θ_min_ = 2.4°
Absorption correction: multi-scan (*SADABS*; Bruker, 2009)	*h* = −10→11
*T*_min_ = 0.693, *T*_max_ = 0.747	*k* = −13→13
31703 measured reflections	*l* = −24→24

### Refinement

**Table:**

Refinement on *F*^2^	Primary atom site location: structure-invariant direct methods
Least-squares matrix: full	Secondary atom site location: difference Fourier map
*R*[*F*^2^ > 2σ(*F*^2^)] = 0.042	Hydrogen site location: inferred from neighbouring sites
*wR*(*F*^2^) = 0.120	H-atom parameters constrained
*S* = 1.02	*w* = 1/[σ^2^(*F*_o_^2^) + (0.0594*P*)^2^ + 0.4278*P*] where *P* = (*F*_o_^2^ + 2*F*_c_^2^)/3
6791 reflections	(Δ/σ)_max_ = 0.001
415 parameters	Δρ_max_ = 0.32 e Å^−^^3^
0 restraints	Δρ_min_ = −0.28 e Å^−^^3^

### Special details

**Table:**

Geometry. All e.s.d.'s (except the e.s.d. in the dihedral angle between two l.s. planes) are estimated using the full covariance matrix. The cell e.s.d.'s are taken into account individually in the estimation of e.s.d.'s in distances, angles and torsion angles; correlations between e.s.d.'s in cell parameters are only used when they are defined by crystal symmetry. An approximate (isotropic) treatment of cell e.s.d.'s is used for estimating e.s.d.'s involving l.s. planes.
Refinement. Refinement of *F*^2^ against all reflections. The weighted *R*-factor *wR* and goodness of fit *S* are based on *F*^2^, conventional *R*-factors *R* are based on *F*, with *F* set to zero for negative *F*^2^. The threshold expression of *F*^2^ > 2σ(*F*^2^) is used only for calculating *R*-factors(gt) *etc*. and is not relevant to the choice of reflections for refinement. *R*-factors based on *F*^2^ are statistically about twice as large as those based on *F*, and *R*- factors based on all data will be even larger.

### Fractional atomic coordinates and isotropic or equivalent isotropic displacement parameters (Å^2^)

**Table:**

	*x*	*y*	*z*	*U*_iso_*/*U*_eq_	
C1	−0.5331 (4)	1.4103 (5)	1.1048 (2)	0.1376 (14)	
H1A	−0.4762	1.3460	1.1375	0.165*	
H1B	−0.5362	1.5009	1.1007	0.165*	
C2	−0.6054 (3)	1.3750 (3)	1.06626 (16)	0.0943 (8)	
H2	−0.6610	1.4419	1.0341	0.113*	
C3	−0.6085 (3)	1.2319 (3)	1.06826 (13)	0.0859 (7)	
H3A	−0.5292	1.1694	1.0966	0.103*	
H3B	−0.7156	1.2276	1.0894	0.103*	
C4	−0.6237 (3)	1.1553 (3)	0.90373 (15)	0.0899 (8)	
H4	−0.6796	1.1473	0.8701	0.108*	
C5	−0.4527 (3)	1.1281 (2)	0.89489 (11)	0.0631 (5)	
C6	−0.4237 (3)	1.1507 (2)	0.95842 (10)	0.0567 (5)	
C7	−0.2677 (3)	1.1323 (2)	0.97123 (10)	0.0626 (5)	
H7	−0.2500	1.1460	1.0140	0.075*	
C8	−0.1409 (3)	1.0934 (2)	0.91886 (9)	0.0561 (5)	
H8	−0.0355	1.0816	0.9260	0.067*	
C9	−0.1670 (2)	1.07082 (18)	0.85424 (9)	0.0483 (4)	
C10	−0.3201 (3)	1.0860 (2)	0.84226 (10)	0.0623 (5)	
H10	−0.3363	1.0688	0.8000	0.075*	
C11	0.0714 (2)	0.7588 (2)	0.81912 (10)	0.0537 (4)	
C12	−0.0059 (3)	0.7344 (2)	0.77026 (12)	0.0680 (6)	
H12	−0.0317	0.7982	0.7317	0.082*	
C13	−0.0443 (3)	0.6161 (3)	0.77871 (15)	0.0810 (7)	
H13	−0.0977	0.6011	0.7460	0.097*	
C14	−0.0053 (3)	0.5185 (2)	0.83494 (16)	0.0806 (7)	
C15	0.0682 (3)	0.5455 (3)	0.88345 (15)	0.0874 (8)	
H15	0.0935	0.4814	0.9219	0.105*	
C16	0.1063 (3)	0.6655 (2)	0.87706 (12)	0.0717 (6)	
H16	0.1543	0.6826	0.9111	0.086*	
C17	−0.0423 (4)	0.3862 (3)	0.8436 (2)	0.1211 (12)	
H17A	−0.1549	0.4054	0.8394	0.182*	
H17B	−0.0214	0.3383	0.8886	0.182*	
H17C	0.0258	0.3305	0.8084	0.182*	
C18	−0.0239 (3)	0.5553 (3)	0.40309 (16)	0.0876 (8)	
H18A	−0.1103	0.5665	0.4393	0.105*	
H18B	0.0332	0.4688	0.3894	0.105*	
C19	0.0165 (3)	0.6589 (2)	0.37214 (12)	0.0632 (5)	
H19	0.1036	0.6438	0.3362	0.076*	
C20	−0.0659 (2)	0.80224 (19)	0.38962 (10)	0.0522 (4)	
H20A	−0.1273	0.8559	0.3527	0.063*	
H20B	−0.1423	0.8019	0.4320	0.063*	
C21	0.2282 (2)	0.96925 (19)	0.36555 (9)	0.0511 (4)	
H21	0.2911	1.0194	0.3383	0.061*	
C22	0.2391 (2)	0.91991 (17)	0.43648 (8)	0.0428 (4)	
C23	0.3346 (2)	0.92338 (17)	0.48490 (8)	0.0442 (4)	
H23	0.4118	0.9684	0.4729	0.053*	
C24	0.3107 (2)	0.85819 (16)	0.55050 (8)	0.0419 (4)	
C25	0.1910 (2)	0.79282 (18)	0.56925 (9)	0.0484 (4)	
H25	0.1768	0.7507	0.6143	0.058*	
C26	0.0952 (2)	0.78944 (18)	0.52319 (9)	0.0488 (4)	
H26	0.0158	0.7467	0.5361	0.059*	
C27	0.1216 (2)	0.85298 (16)	0.45583 (8)	0.0407 (4)	
C28	0.5359 (2)	0.58393 (18)	0.63513 (9)	0.0463 (4)	
C29	0.5396 (2)	0.5001 (2)	0.58778 (10)	0.0552 (5)	
H29	0.5773	0.5188	0.5411	0.066*	
C30	0.4874 (3)	0.3886 (2)	0.60989 (11)	0.0619 (5)	
H30	0.4889	0.3330	0.5775	0.074*	
C31	0.4327 (2)	0.3571 (2)	0.67903 (11)	0.0590 (5)	
C32	0.4335 (3)	0.4411 (2)	0.72592 (11)	0.0726 (6)	
H32	0.3994	0.4207	0.7728	0.087*	
C33	0.4837 (3)	0.5539 (2)	0.70484 (10)	0.0665 (6)	
H33	0.4825	0.6095	0.7371	0.080*	
C34	0.3732 (3)	0.2362 (3)	0.70195 (15)	0.0841 (7)	
H34A	0.2562	0.2675	0.7115	0.126*	
H34B	0.4174	0.1867	0.7432	0.126*	
H34C	0.4081	0.1772	0.6658	0.126*	
N1	−0.5718 (2)	1.18978 (19)	0.99908 (10)	0.0694 (5)	
N2	−0.6939 (2)	1.1936 (2)	0.96546 (12)	0.0855 (6)	
N3	−0.03312 (19)	1.03964 (16)	0.79882 (7)	0.0523 (4)	
H3N	−0.0594	1.0518	0.7611	0.063*	
N4	0.05029 (18)	0.86568 (15)	0.39871 (7)	0.0470 (3)	
N5	0.1175 (2)	0.93510 (16)	0.34324 (7)	0.0521 (4)	
N6	0.40966 (19)	0.85977 (15)	0.60082 (7)	0.0487 (4)	
H6N	0.3615	0.8700	0.6388	0.058*	
O1	0.22363 (18)	0.91011 (16)	0.73713 (7)	0.0677 (4)	
O2	0.20026 (17)	0.91093 (16)	0.86333 (7)	0.0688 (4)	
O3	0.67127 (17)	0.72657 (15)	0.53792 (8)	0.0677 (4)	
O4	0.6511 (2)	0.76466 (16)	0.66010 (8)	0.0748 (4)	
S1	0.12954 (6)	0.90722 (5)	0.80483 (2)	0.05223 (14)	
S2	0.58344 (6)	0.73592 (5)	0.60662 (2)	0.05151 (14)	

### Atomic displacement parameters (Å^2^)

**Table:**

	*U*^11^	*U*^22^	*U*^33^	*U*^12^	*U*^13^	*U*^23^
C1	0.092 (2)	0.163 (4)	0.149 (3)	−0.015 (2)	0.001 (2)	−0.071 (3)
C2	0.0788 (17)	0.105 (2)	0.0888 (19)	−0.0081 (15)	0.0001 (15)	−0.0433 (17)
C3	0.0829 (16)	0.099 (2)	0.0613 (14)	−0.0196 (14)	0.0163 (12)	−0.0202 (13)
C4	0.0640 (14)	0.117 (2)	0.0809 (17)	−0.0096 (14)	−0.0194 (13)	−0.0214 (16)
C5	0.0589 (12)	0.0669 (13)	0.0564 (12)	−0.0073 (10)	−0.0145 (9)	−0.0089 (10)
C6	0.0649 (12)	0.0509 (11)	0.0464 (10)	−0.0117 (9)	−0.0036 (9)	−0.0046 (8)
C7	0.0738 (13)	0.0749 (14)	0.0397 (10)	−0.0214 (11)	−0.0072 (9)	−0.0148 (9)
C8	0.0629 (11)	0.0668 (12)	0.0418 (10)	−0.0212 (10)	−0.0109 (8)	−0.0104 (9)
C9	0.0598 (11)	0.0455 (10)	0.0361 (9)	−0.0112 (8)	−0.0090 (8)	−0.0054 (7)
C10	0.0628 (12)	0.0757 (14)	0.0446 (10)	−0.0091 (10)	−0.0179 (9)	−0.0141 (9)
C11	0.0500 (10)	0.0561 (11)	0.0461 (10)	−0.0064 (8)	−0.0039 (8)	−0.0082 (8)
C12	0.0773 (14)	0.0674 (14)	0.0587 (12)	−0.0190 (11)	−0.0152 (11)	−0.0090 (10)
C13	0.0810 (16)	0.0753 (16)	0.0906 (18)	−0.0244 (13)	−0.0089 (14)	−0.0253 (14)
C14	0.0586 (13)	0.0602 (14)	0.109 (2)	−0.0098 (11)	0.0117 (13)	−0.0202 (14)
C15	0.0765 (16)	0.0666 (15)	0.0922 (19)	−0.0075 (13)	−0.0019 (14)	0.0186 (13)
C16	0.0676 (13)	0.0738 (15)	0.0627 (13)	−0.0123 (11)	−0.0157 (11)	0.0060 (11)
C17	0.096 (2)	0.0656 (17)	0.186 (4)	−0.0242 (15)	0.022 (2)	−0.0225 (19)
C18	0.0931 (18)	0.0565 (14)	0.113 (2)	−0.0224 (13)	−0.0220 (16)	−0.0035 (13)
C19	0.0685 (13)	0.0618 (13)	0.0663 (13)	−0.0244 (10)	−0.0145 (10)	−0.0131 (10)
C20	0.0522 (10)	0.0506 (10)	0.0589 (11)	−0.0191 (8)	−0.0206 (9)	−0.0003 (8)
C21	0.0652 (11)	0.0579 (11)	0.0378 (9)	−0.0308 (9)	−0.0112 (8)	0.0015 (8)
C22	0.0522 (9)	0.0408 (9)	0.0368 (8)	−0.0165 (7)	−0.0081 (7)	−0.0034 (7)
C23	0.0528 (10)	0.0440 (9)	0.0401 (9)	−0.0199 (8)	−0.0102 (7)	−0.0032 (7)
C24	0.0502 (9)	0.0379 (8)	0.0354 (8)	−0.0087 (7)	−0.0091 (7)	−0.0069 (6)
C25	0.0555 (10)	0.0491 (10)	0.0356 (9)	−0.0142 (8)	−0.0053 (7)	0.0016 (7)
C26	0.0500 (10)	0.0489 (10)	0.0469 (10)	−0.0193 (8)	−0.0063 (8)	0.0025 (8)
C27	0.0449 (9)	0.0374 (8)	0.0393 (8)	−0.0107 (7)	−0.0093 (7)	−0.0050 (7)
C28	0.0499 (10)	0.0464 (9)	0.0412 (9)	−0.0119 (8)	−0.0129 (7)	−0.0018 (7)
C29	0.0680 (12)	0.0533 (11)	0.0410 (9)	−0.0129 (9)	−0.0123 (9)	−0.0054 (8)
C30	0.0750 (13)	0.0516 (11)	0.0620 (12)	−0.0168 (10)	−0.0230 (10)	−0.0072 (9)
C31	0.0508 (11)	0.0517 (11)	0.0723 (13)	−0.0131 (9)	−0.0212 (10)	0.0055 (10)
C32	0.0875 (16)	0.0749 (15)	0.0500 (12)	−0.0296 (13)	0.0003 (11)	0.0029 (10)
C33	0.0954 (16)	0.0643 (13)	0.0425 (10)	−0.0284 (12)	−0.0078 (10)	−0.0098 (9)
C34	0.0725 (15)	0.0758 (16)	0.109 (2)	−0.0343 (13)	−0.0311 (14)	0.0193 (14)
N1	0.0679 (11)	0.0688 (12)	0.0599 (11)	−0.0126 (9)	0.0029 (9)	−0.0103 (9)
N2	0.0631 (12)	0.0942 (16)	0.0848 (15)	−0.0095 (11)	−0.0043 (11)	−0.0130 (12)
N3	0.0643 (9)	0.0581 (9)	0.0312 (7)	−0.0139 (8)	−0.0103 (7)	−0.0054 (6)
N4	0.0537 (8)	0.0483 (8)	0.0432 (8)	−0.0204 (7)	−0.0141 (6)	0.0000 (6)
N5	0.0657 (10)	0.0584 (9)	0.0375 (8)	−0.0261 (8)	−0.0145 (7)	0.0008 (7)
N6	0.0615 (9)	0.0488 (8)	0.0349 (7)	−0.0134 (7)	−0.0111 (6)	−0.0074 (6)
O1	0.0662 (9)	0.0856 (10)	0.0476 (8)	−0.0247 (8)	0.0051 (6)	−0.0126 (7)
O2	0.0656 (9)	0.0885 (11)	0.0535 (8)	−0.0167 (8)	−0.0229 (7)	−0.0129 (7)
O3	0.0603 (8)	0.0687 (9)	0.0640 (9)	−0.0187 (7)	0.0032 (7)	0.0018 (7)
O4	0.0865 (11)	0.0710 (10)	0.0846 (11)	−0.0340 (8)	−0.0475 (9)	0.0018 (8)
S1	0.0529 (3)	0.0649 (3)	0.0371 (2)	−0.0147 (2)	−0.00791 (19)	−0.0086 (2)
S2	0.0554 (3)	0.0524 (3)	0.0496 (3)	−0.0189 (2)	−0.0165 (2)	−0.0002 (2)

### Geometric parameters (Å, º)

**Table:**

C1—C2	1.252 (4)	C20—N4	1.451 (2)
C1—H1A	0.9300	C20—H20A	0.9700
C1—H1B	0.9300	C20—H20B	0.9700
C2—C3	1.502 (4)	C21—N5	1.317 (2)
C2—H2	0.9300	C21—C22	1.417 (2)
C3—N1	1.450 (3)	C21—H21	0.9300
C3—H3A	0.9700	C22—C23	1.402 (2)
C3—H3B	0.9700	C22—C27	1.404 (2)
C4—N2	1.317 (3)	C23—C24	1.372 (2)
C4—C5	1.417 (3)	C23—H23	0.9300
C4—H4	0.9300	C24—C25	1.409 (2)
C5—C6	1.400 (3)	C24—N6	1.448 (2)
C5—C10	1.406 (3)	C25—C26	1.367 (2)
C6—N1	1.364 (3)	C25—H25	0.9300
C6—C7	1.388 (3)	C26—C27	1.398 (2)
C7—C8	1.368 (3)	C26—H26	0.9300
C7—H7	0.9300	C27—N4	1.359 (2)
C8—C9	1.410 (2)	C28—C29	1.376 (3)
C8—H8	0.9300	C28—C33	1.384 (3)
C9—C10	1.365 (3)	C28—S2	1.7592 (18)
C9—N3	1.435 (2)	C29—C30	1.375 (3)
C10—H10	0.9300	C29—H29	0.9300
C11—C16	1.380 (3)	C30—C31	1.382 (3)
C11—C12	1.384 (3)	C30—H30	0.9300
C11—S1	1.762 (2)	C31—C32	1.383 (3)
C12—C13	1.370 (3)	C31—C34	1.503 (3)
C12—H12	0.9300	C32—C33	1.375 (3)
C13—C14	1.383 (4)	C32—H32	0.9300
C13—H13	0.9300	C33—H33	0.9300
C14—C15	1.369 (4)	C34—H34A	0.9600
C14—C17	1.506 (4)	C34—H34B	0.9600
C15—C16	1.390 (4)	C34—H34C	0.9600
C15—H15	0.9300	N1—N2	1.355 (3)
C16—H16	0.9300	N3—S1	1.6219 (16)
C17—H17A	0.9600	N3—H3N	0.7979
C17—H17B	0.9600	N4—N5	1.363 (2)
C17—H17C	0.9600	N6—S2	1.6396 (16)
C18—C19	1.280 (3)	N6—H6N	0.7925
C18—H18A	0.9300	O1—S1	1.4337 (14)
C18—H18B	0.9300	O2—S1	1.4253 (14)
C19—C20	1.493 (3)	O3—S2	1.4283 (15)
C19—H19	0.9300	O4—S2	1.4260 (14)
			
C2—C1—H1A	120.0	N5—C21—H21	124.3
C2—C1—H1B	120.0	C22—C21—H21	124.3
H1A—C1—H1B	120.0	C23—C22—C27	120.20 (15)
C1—C2—C3	124.6 (4)	C23—C22—C21	135.59 (16)
C1—C2—H2	117.7	C27—C22—C21	104.21 (15)
C3—C2—H2	117.7	C24—C23—C22	117.89 (15)
N1—C3—C2	111.3 (2)	C24—C23—H23	121.1
N1—C3—H3A	109.4	C22—C23—H23	121.1
C2—C3—H3A	109.4	C23—C24—C25	121.15 (15)
N1—C3—H3B	109.4	C23—C24—N6	118.47 (15)
C2—C3—H3B	109.4	C25—C24—N6	120.37 (14)
H3A—C3—H3B	108.0	C26—C25—C24	122.00 (16)
N2—C4—C5	111.7 (2)	C26—C25—H25	119.0
N2—C4—H4	124.2	C24—C25—H25	119.0
C5—C4—H4	124.2	C25—C26—C27	117.11 (16)
C6—C5—C10	119.11 (19)	C25—C26—H26	121.4
C6—C5—C4	104.1 (2)	C27—C26—H26	121.4
C10—C5—C4	136.8 (2)	N4—C27—C26	131.53 (16)
N1—C6—C7	131.43 (19)	N4—C27—C22	106.84 (14)
N1—C6—C5	106.56 (19)	C26—C27—C22	121.63 (15)
C7—C6—C5	122.02 (18)	C29—C28—C33	120.04 (18)
C8—C7—C6	117.81 (18)	C29—C28—S2	120.13 (14)
C8—C7—H7	121.1	C33—C28—S2	119.67 (14)
C6—C7—H7	121.1	C30—C29—C28	119.62 (18)
C7—C8—C9	121.28 (19)	C30—C29—H29	120.2
C7—C8—H8	119.4	C28—C29—H29	120.2
C9—C8—H8	119.4	C29—C30—C31	121.56 (19)
C10—C9—C8	120.84 (17)	C29—C30—H30	119.2
C10—C9—N3	119.27 (16)	C31—C30—H30	119.2
C8—C9—N3	119.79 (17)	C30—C31—C32	117.79 (19)
C9—C10—C5	118.93 (18)	C30—C31—C34	120.7 (2)
C9—C10—H10	120.5	C32—C31—C34	121.5 (2)
C5—C10—H10	120.5	C33—C32—C31	121.6 (2)
C16—C11—C12	120.0 (2)	C33—C32—H32	119.2
C16—C11—S1	120.87 (17)	C31—C32—H32	119.2
C12—C11—S1	119.12 (16)	C32—C33—C28	119.35 (19)
C13—C12—C11	119.9 (2)	C32—C33—H33	120.3
C13—C12—H12	120.0	C28—C33—H33	120.3
C11—C12—H12	120.0	C31—C34—H34A	109.5
C12—C13—C14	121.3 (3)	C31—C34—H34B	109.5
C12—C13—H13	119.4	H34A—C34—H34B	109.5
C14—C13—H13	119.4	C31—C34—H34C	109.5
C15—C14—C13	118.1 (2)	H34A—C34—H34C	109.5
C15—C14—C17	120.2 (3)	H34B—C34—H34C	109.5
C13—C14—C17	121.7 (3)	N2—N1—C6	111.58 (18)
C14—C15—C16	122.1 (2)	N2—N1—C3	120.2 (2)
C14—C15—H15	119.0	C6—N1—C3	128.1 (2)
C16—C15—H15	119.0	C4—N2—N1	106.1 (2)
C11—C16—C15	118.6 (2)	C9—N3—S1	123.01 (12)
C11—C16—H16	120.7	C9—N3—H3N	114.5
C15—C16—H16	120.7	S1—N3—H3N	110.5
C14—C17—H17A	109.5	C27—N4—N5	111.13 (13)
C14—C17—H17B	109.5	C27—N4—C20	128.27 (15)
H17A—C17—H17B	109.5	N5—N4—C20	120.10 (14)
C14—C17—H17C	109.5	C21—N5—N4	106.45 (14)
H17A—C17—H17C	109.5	C24—N6—S2	118.67 (11)
H17B—C17—H17C	109.5	C24—N6—H6N	114.2
C19—C18—H18A	120.0	S2—N6—H6N	108.4
C19—C18—H18B	120.0	O2—S1—O1	118.84 (9)
H18A—C18—H18B	120.0	O2—S1—N3	109.12 (8)
C18—C19—C20	124.7 (2)	O1—S1—N3	104.33 (8)
C18—C19—H19	117.6	O2—S1—C11	108.08 (9)
C20—C19—H19	117.6	O1—S1—C11	107.75 (9)
N4—C20—C19	111.89 (16)	N3—S1—C11	108.31 (9)
N4—C20—H20A	109.2	O4—S2—O3	120.18 (10)
C19—C20—H20A	109.2	O4—S2—N6	105.67 (9)
N4—C20—H20B	109.2	O3—S2—N6	106.80 (8)
C19—C20—H20B	109.2	O4—S2—C28	108.23 (9)
H20A—C20—H20B	107.9	O3—S2—C28	108.43 (9)
N5—C21—C22	111.35 (15)	N6—S2—C28	106.79 (8)
			
C1—C2—C3—N1	−132.5 (3)	C29—C30—C31—C34	178.9 (2)
N2—C4—C5—C6	1.2 (3)	C30—C31—C32—C33	1.3 (3)
N2—C4—C5—C10	−179.8 (3)	C34—C31—C32—C33	−178.2 (2)
C10—C5—C6—N1	−179.90 (19)	C31—C32—C33—C28	−0.5 (4)
C4—C5—C6—N1	−0.6 (2)	C29—C28—C33—C32	−1.0 (3)
C10—C5—C6—C7	−0.3 (3)	S2—C28—C33—C32	174.52 (18)
C4—C5—C6—C7	178.9 (2)	C7—C6—N1—N2	−179.6 (2)
N1—C6—C7—C8	−179.2 (2)	C5—C6—N1—N2	0.0 (2)
C5—C6—C7—C8	1.3 (3)	C7—C6—N1—C3	4.1 (4)
C6—C7—C8—C9	−0.8 (3)	C5—C6—N1—C3	−176.4 (2)
C7—C8—C9—C10	−0.6 (3)	C2—C3—N1—N2	−100.4 (3)
C7—C8—C9—N3	175.55 (18)	C2—C3—N1—C6	75.7 (3)
C8—C9—C10—C5	1.6 (3)	C5—C4—N2—N1	−1.2 (3)
N3—C9—C10—C5	−174.58 (18)	C6—N1—N2—C4	0.8 (3)
C6—C5—C10—C9	−1.1 (3)	C3—N1—N2—C4	177.5 (2)
C4—C5—C10—C9	179.9 (3)	C10—C9—N3—S1	−124.00 (18)
C16—C11—C12—C13	−1.5 (3)	C8—C9—N3—S1	59.8 (2)
S1—C11—C12—C13	176.62 (18)	C26—C27—N4—N5	−179.05 (18)
C11—C12—C13—C14	−0.9 (4)	C22—C27—N4—N5	1.07 (19)
C12—C13—C14—C15	2.2 (4)	C26—C27—N4—C20	−7.3 (3)
C12—C13—C14—C17	−178.1 (2)	C22—C27—N4—C20	172.87 (17)
C13—C14—C15—C16	−1.0 (4)	C19—C20—N4—C27	−81.8 (2)
C17—C14—C15—C16	179.2 (2)	C19—C20—N4—N5	89.4 (2)
C12—C11—C16—C15	2.6 (3)	C22—C21—N5—N4	1.4 (2)
S1—C11—C16—C15	−175.51 (17)	C27—N4—N5—C21	−1.5 (2)
C14—C15—C16—C11	−1.3 (4)	C20—N4—N5—C21	−174.08 (16)
C18—C19—C20—N4	129.8 (2)	C23—C24—N6—S2	−90.15 (17)
N5—C21—C22—C23	178.45 (19)	C25—C24—N6—S2	90.84 (18)
N5—C21—C22—C27	−0.7 (2)	C9—N3—S1—O2	−56.27 (17)
C27—C22—C23—C24	0.9 (2)	C9—N3—S1—O1	175.74 (14)
C21—C22—C23—C24	−178.2 (2)	C9—N3—S1—C11	61.16 (16)
C22—C23—C24—C25	−1.6 (3)	C16—C11—S1—O2	−4.08 (19)
C22—C23—C24—N6	179.38 (14)	C12—C11—S1—O2	177.84 (16)
C23—C24—C25—C26	0.9 (3)	C16—C11—S1—O1	125.51 (17)
N6—C24—C25—C26	179.90 (16)	C12—C11—S1—O1	−52.57 (18)
C24—C25—C26—C27	0.5 (3)	C16—C11—S1—N3	−122.18 (17)
C25—C26—C27—N4	178.88 (17)	C12—C11—S1—N3	59.74 (18)
C25—C26—C27—C22	−1.3 (3)	C24—N6—S2—O4	−179.54 (13)
C23—C22—C27—N4	−179.55 (15)	C24—N6—S2—O3	51.40 (15)
C21—C22—C27—N4	−0.22 (19)	C24—N6—S2—C28	−64.46 (14)
C23—C22—C27—C26	0.6 (3)	C29—C28—S2—O4	−150.75 (16)
C21—C22—C27—C26	179.88 (16)	C33—C28—S2—O4	33.78 (19)
C33—C28—C29—C30	1.6 (3)	C29—C28—S2—O3	−18.86 (18)
S2—C28—C29—C30	−173.83 (15)	C33—C28—S2—O3	165.67 (17)
C28—C29—C30—C31	−0.8 (3)	C29—C28—S2—N6	95.90 (16)
C29—C30—C31—C32	−0.6 (3)	C33—C28—S2—N6	−79.58 (17)

### Hydrogen-bond geometry (Å, º)

**Table:**

*D*—H···*A*	*D*—H	H···*A*	*D*···*A*	*D*—H···*A*
N6—H6*N*···O1	0.79	2.11	2.900 (2)	176
N3—H3*N*···N5^i^	0.80	2.19	2.983 (2)	175
C21—H21···O4^ii^	0.93	2.49	3.245 (2)	138
C7—H7···O2^iii^	0.93	2.48	3.358 (2)	158

Symmetry codes: (i) −*x*, −*y*+2, −*z*+1; (ii) −*x*+1, −*y*+2, −*z*+1; (iii) −*x*, −*y*+2, −*z*+2.
